# Time of Application of Desiccant Herbicides Affects Photosynthetic Pigments, Physiological Indicators, and the Quality of Cowpea Seeds

**DOI:** 10.3390/jox14030074

**Published:** 2024-09-19

**Authors:** Ester dos Santos Coêlho, João Everthon da Silva Ribeiro, Welder de Araújo Rangel Lopes, Anna Kézia Soares de Oliveira, Pablo Henrique de Almeida Oliveira, Gisele Lopes dos Santos, Ewerton da Silva Barbosa, Valécia Nogueira Santos e Silva, Hamurábi Anizio Lins, Clarisse Pereira Benedito, Lindomar Maria da Silveira, Antonio Cesar de Araujo Filho, Daniel Valadão Silva, Aurélio Paes Barros Júnior

**Affiliations:** 1 Agricultural Sciences Center, Federal Rural University of the Semi-Arid Region, Mossoró 59625-900, Brazil; estersantos12@hotmail.com (E.d.S.C.); welder.lopes@hotmail.com (W.d.A.R.L.); annakezia@outlook.com (A.K.S.d.O.); pabloalmeidaagro@gmail.com (P.H.d.A.O.); gisele1612@gmail.com (G.L.d.S.); valecia.santos@gmail.com (V.N.S.e.S.); hamurabi_a_@hotmail.com (H.A.L.); clarisse@ufersa.edu.br (C.P.B.); lindomarmaria@ufersa.edu.br (L.M.d.S.); cesar3aeg@gmail.com (A.C.d.A.F.); daniel.valadao@ufersa.edu.br (D.V.S.); aurelio.barros@ufersa.edu.br (A.P.B.J.); 2 Agricultural Sciences Center, Federal University of Paraíba, Areia 58397-000, Brazil; ewertonsilva07@gmail.com

**Keywords:** *Vigna unguiculata* (L.) Walp., physiological quality, germination, toxicity

## Abstract

Chemical desiccation is widely used in agriculture to anticipate harvest and mitigate the effects of adverse environmental conditions. It is applied to both grains and seeds. Although this practice is widely used, there are still significant gaps in understanding the effects of different herbicide application times on seed quality and plant physiological responses. The objective of this study was to evaluate the effects of different herbicide application times on cowpea, focusing on seed quality, physiological responses, and biochemical composition, including chlorophylls, carotenoids, sugars, and proline, under nocturnal desiccation. In the first experiment, eight herbicides and two mixtures were applied at night: diquat, flumioxazin, diquat + flumioxazin, glufosinate ammonium, saflufenacil, carfentrazone, diquat + carfentrazone, atrazine, and glyphosate. All of the tested herbicides caused a reduction in normal seedling formation, with the diquat + carfentrazone combination resulting in 100% abnormal seedlings. A significant decrease in chlorophyll levels (chlorophyll a: 63.5%, chlorophyll b: 50.2%) was observed using diquat, which indicates damage to photosynthetic processes, while the carotenoid content increased. Total soluble sugars and proline were also negatively impacted, reflecting physiological stress and metabolic changes in seedlings. In the second experiment, three application times were tested with diquat, diquat + flumioxazin, and diquat + carfentrazone. Nocturnal application showed the most significant reduction in chlorophyll levels and increased carotenoid levels. Application at noon and late afternoon also significantly changed the soluble sugar and proline levels. These results indicate that the herbicide application time directly influences the seeds’ physiological quality.

## 1. Introduction

The nutritional relevance and productive potential of cowpea [*Vigna unguiculata* (L.) Walp.] make this crop an essential source of vegetable protein, consumed and cultivated in various parts of the world [1]. In Brazil, it was traditionally cultivated in the North and Northeast regions; however, in recent years, cultivated areas have expanded throughout the country with technological improvements in management [2]. Cowpea is a legume with broad importance in human nutrition and is considered a low-cost protein source [3]. It is a food rich in vitamins, minerals, fiber, carbohydrates, lipids, and proteins [4]. It also has phenolic compounds, antioxidant, anti-inflammatory, and antihypertensive properties [5]. In the current agricultural scenario, cowpea planting areas are increasingly being expanded [6], and this has been attributed to the great diversity of varieties with more significant yield potential, better grain and seed quality, and early maturation cycles [7].

The high productive potential of cowpea is associated with its ideal harvest point, which is to obtain quality seeds with high germination capacity and vigor [8]. Given this aspect, it is essential to consider the maturation phase of the plants so that the harvest season is carried out after physiological maturity [9]. The importance of defining the harvest time should also be chosen to reduce the exposure time in the field, as late harvests can expose the plants to adverse environmental conditions and the occurrence of pests and diseases, thus altering the final quality of the seeds [10]. In the search for more productive cultivars with shorter exposure time in the field, using herbicides has proven to be an efficient strategy not only for weed control but also for anticipating maturation and enabling mechanized harvesting. In addition to controlling competition with weeds, desiccating herbicides accelerate the senescence process of cultivated plants, allowing for faster harvesting and lower risks of losses due to environmental factors [11].

Using herbicides is of great importance for the preharvest of cowpea, as they cause shorter cycles that provide competitiveness in the sector and prevent productivity losses [12]. In addition, with the application of these herbicides, uniformity in the maturation of pods can be obtained without inducing dehiscence [2]. For this, it should be considered that herbicides with greater translocation may cause a greater possibility of additional damage to the seeds [13]. Preharvest desiccation is a common practice for crops such as soybeans [14], canola [15], wheat [9], and common beans [16]. For most crops, herbicides are registered for desiccation; however, in the cowpea crop, there are no registered products [17].

The choice of herbicide to be used is based on its translocation, since, for desiccation, it is ideal that the herbicide is in contact with fast action and low mobility in the plant [18]. In order to be efficient in the application of these products, it is essential to consider factors such as the herbicide’s mode of action, environmental conditions, time of application, and phenological stage of the crop to avoid the occurrence of residues that compromise the quality of seeds and grains [19].

Environmental conditions throughout the day can interfere positively or negatively with the action of herbicides, making it complex to make decisions about the best time for application [20]. According to Johnston et al. (2018) [21], translocation and phytotoxicity are processes affected by the time of herbicide application. Environmental factors can alter the viscosity of lipids in the leaf cuticle, directly influencing the effect of herbicides [21,22]. The oscillation of temperature throughout the day causes changes in the hydration of the cuticle and thus causes changes in the physical process of diffusion of herbicides [23].

In addition to these factors, a high relative humidity of the air is directly associated with the water status of the plant, which regulates the stomata’s opening and can intensify herbicides’ absorption and translocation [24]. On the other hand, a lower relative humidity of the air increases the possibility of evaporation of droplets [25]. Changes in luminosity can also influence the translocation of herbicides through the depth effect that enhances the action of herbicides; they can also provide photodegradation of specific herbicides when exposed to conditions of intense luminosity [25].

In the search for information about herbicides applied for desiccation in cowpea, it is necessary to carry out studies that provide information about the activity of herbicides due to the time of application, the effects caused on the seeds, and the physiological mechanisms that can occur to mitigate greater translocation and phytotoxicity [21,26]. Thus, the hypothesis was tested that herbicides applied at different times throughout the day cause changes in the physiological quality of cowpea seeds. Thus, the objectives of this work were as follows: (1) to identify the ideal time for the application of herbicides in cowpea; (2) to investigate the effects of different herbicide application schedules on cowpea seed quality and physiology; (3) to analyze the content of chlorophylls, carotenoids, total soluble sugars, and proline in cowpea seedlings submitted to chemical desiccation at night.

## 2. Materials and Methods

### 2.1. Characterization of the Study Area and Field Conduction

This study was carried out at the Didactic Garden of the Agricultural Sciences Center, Federal Rural University of the Semi-Arid Region (UFERSA) in Mossoró-RN, Brazil. The first study (Experiment I) took place between June and August 2021, while the second study (Experiment II) was conducted from October to December 2021. According to the Köppen climate classification, the region has a hot semi-arid climate (BSh) [27], with an average annual temperature of 27.5 °C and an average annual rainfall of 670 mm. During the study period, the accumulated rainfall was 6.35 mm in Experiment I and 78.4 mm in Experiment II, with average temperatures of 28.5 °C and 29.6 °C, respectively. Meteorological data were obtained from the Automatic Meteorological Station of the Engineering Center at UFERSA, Mossoró-RN, Brazil.

The soil in the experimental site is categorized as eutrophic red-yellow Ultisol, based on the classification by the Empresa Brasileira de Pesquisa Agropecuária [28]. The texture of red-yellow Ultisols typically ranges from sandy loam to clay loam, with a predominance of fine particles such as silt and clay. These soils have a well-developed structure, often crumbly and friable in the upper layers, allowing for good root penetration and water infiltration. Prior to the commencement of the experiments, 15 individual soil samples were gathered at depths of 20 cm and 40 cm. These samples were subsequently merged to create a composite sample, the findings of which are detailed in Table 1.

The experimental plots were arranged in four rows, each measuring 4.0 m in length, with a spacing of 0.5 m between rows and 0.2 m between individual plants. Four seeds were planted in each hole, and thinning was performed 14 days after planting, resulting in two plants remaining per hole. The cultivar utilized was BRS Tumucumaque, recognized for its semi-erect growth habit, 70-to-75-day development cycle, and robust and productive potential. The seeds were obtained from the germplasm bank of UFERSA, Mossoró, Brazil.

Cultural practices were carried out using technical recommendations for the crop [29]. Thirty days after sowing, topdressing fertilization was carried out, providing 30 kg of nitrogen (N) per hectare, 15 kg of phosphorus (P) per hectare, and 10 kg of potassium (K) per hectare. Urea (containing 45% N), mono ammonium phosphate (MAP) (with 54% P_2_O_5_), and potassium chloride (KCl) (with 60% K_2_O) were used as nutrient sources and were applied according to the crop’s nutritional needs. Weed management was performed by manual weeding as needed when the infestation reached levels that could compromise crop development. Pest control was performed with two applications of the insecticide Connect at a rate of 700 mL per hectare. The product was applied to control insect pests that could affect crop yield based on technical recommendations for integrated pest management.

### 2.2. Experiment I

#### 2.2.1. Experimental Design and Treatments

The experimental setup utilized a randomized block design consisting of three replications and ten different treatments: diquat, flumioxazin, a combination of diquat and flumioxazin, glufosinate ammonium, saflufenacil, carfentrazone, a mix of diquat and carfentrazone, atrazine, glyphosate, and a control group with no treatment applied. The characteristics of the herbicides employed in the experiment are detailed in Table 2. The doses of desiccant herbicides were determined based on the manufacturers’ recommendations, considering the characteristics of each product and the conditions of the crop under study.

#### 2.2.2. Herbicide Application

The herbicides were used 65 days after sowing (DAS) using a CO_2_-pressurized knapsack sprayer fitted with two TT11002 air induction spray tips (Herbictat, Catanduva, SP, Brazil), operating at a pressure of 3 bar. The choice of these air induction tips aims to reduce drift, promote more uniform droplet deposition, and improve the efficiency of herbicide application. The application took place between 7 p.m. and 8 p.m., utilizing a spray volume of 200 L per hectare. During this process, the recorded climatic conditions included a wind speed of 3.24 m/s and a relative humidity of 71.17%, as measured by the Automatic Meteorological Station at UFERSA.

### 2.3. Experiment II

#### 2.3.1. Experimental Design and Treatments

The experimental setup utilized a randomized block design structured as a double factorial scheme, incorporating an additional treatment, represented as (3 × 3 + 1), with three replications. The treatments consisted of various combinations of the herbicides diquat, carfentrazone, and flumioxazin, categorized as follows: T1 included diquat, T2 combined diquat with carfentrazone, and T3 combined diquat with flumioxazin. Additionally, three application times were tested: 6 a.m., 12 p.m., and 6 p.m. A control, which did not receive any herbicide application, served as the additional treatment.

#### 2.3.2. Herbicide Application

The herbicides were applied 71 days after sowing, using a CO_2_-pressurized knapsack sprayer equipped with two TT11002 air induction spray tips (Herbictat, Catanduva, SP, Brazil), operating at a pressure of 3 bar. The mixture was applied at a volume of 200 L per hectare. At the time of application, the recorded climatic conditions included wind speeds of 1.78 m/s at 6 a.m., 3.51 m/s at noon, and 3.70 m/s at 6 p.m., along with relative humidity levels of 84.2% at 6 a.m., 40.0% at 12 p.m., and 65.58% at 6 p.m. These climatic data were obtained from the Automatic Meteorological Station at UFERSA.

### 2.4. Harvesting and Preparation of Seeds

After applying the treatments, the plants’ defoliation and desiccation were monitored daily. The pods were collected once plant desiccation was visually complete before reaching critical levels of deterioration and then taken to the laboratory, where they were manually threshed. The moisture content in the seeds was determined immediately after harvesting each treatment. Subsequently, the moisture content of the seeds was determined using two replicates of 25 in an oven at 105 ± 3 °C for 24 h, where the moisture content of the seeds was determined [30]. The seeds that presented a moisture content less than or equal to 13.0% were stored in plastic bottles in a refrigerated chamber (17 °C; ±40% of relative humidity) at the Seed Analysis Laboratory of the Federal Rural University of the Semi-Arid (UFERSA) for the analysis of germination, vigor, and initial growth of seedlings.

### 2.5. Variables Analyzed

#### 2.5.1. Experiment I

##### Germination Test

For the germination, four sets of 50 seeds each were tested. The seeds were arranged on moistened germination paper, with the paper rolls containing 2.5 times the weight of the dry paper in distilled water. Each treatment and its corresponding replications were placed in plastic bags and incubated at 25 °C with a 12 h photoperiod in a germination chamber. After eight days, the germination percentage was calculated by counting the number of normal seedlings that had developed [30].

##### First Germination Count

The first germination count (FGC) represented the percentage of healthy seedlings that had emerged [30], with measurements taken on the fifth day following sowing.

##### Average Germination Speed

The average germination rate (AGR) was calculated based on daily tallies of germinated seeds, using the formula put forth by Labouriau and Valadares in 1976 [31]:

$$AGS=\frac{1}{t}$$

where *t* = average germination time.

##### Germination Speed Index

The germination speed index (GSI) was established through daily tallies of healthy seedlings. These counts were conducted over five days, from the third to the eighth day post-sowing, and the index was computed using the equation suggested by Maguire in 1962 [32]:

$$GSI=\frac{G1}{n1}+\frac{G2}{n2}+\ldots +\frac{Gi}{ni}$$

where *GSI* = seedling emergence speed index; *G* = number of seeds germinated each day; *n* = number of days elapsed from sowing to the last count.

##### Length and Dry Mass of Root and Shoot

Root length (RL), shoot length (SL), root dry mass (RDM), and shoot dry mass (SDM) measurements were performed at the end of the germination test (08 days after sowing). The cotyledons were removed, and the shoots and primary roots of the normal seedlings were measured using a ruler graduated in centimeters, with the results expressed in cm seedling^−1^.

Normal seedlings were packed in paper bags and placed in an oven with forced air circulation at 65 °C for 72 h. Shoot and root dry mass was determined with an analytical balance (0.0001 g) (Bel M214-AIH, Piracicaba, SP, Brazil). The results were expressed in g seedling^−1^.

##### Electrical Conductivity

Electrical conductivity was measured using four replicates of 50 seeds for each treatment, which were placed in plastic cups (200 mL capacity) and weighed on a 0.01 g precision scale. After weighing, the seeds were soaked in 75 mL of distilled water and kept for 24 h at 30 °C [33]. After this period, electrical conductivity was measured using a benchtop conductivity meter and the results were expressed in μS cm^−1^ g^−1^ of seed.

##### Accelerated Aging Test

For the accelerated aging test, 250 seeds from each treatment were placed on aluminum screens in gearbox with 40 mL of distilled water. The boxes were closed and kept in a Biochemical Oxygen Demand (347-CD, Fanen, Guarulhos, SP, Brazil) incubator for 48 h at a temperature of 42 °C [33]. Subsequently, the germination test was carried out, with four replicates of 50 seeds, to evaluate the percentages of normal seedlings on the fifth day.

##### Preparation of the Plant Extract

In each replication, ten samples of healthy seedlings were chosen, placed in plastic bags, and stored in a freezer at −10 °C. To prepare the plant extract for the biochemical assays, 0.2 g of fresh seedling mass was measured and transferred to sealed tubes, to which 3 mL of 60% alcohol was added. The plant material was then macerated, and the tubes were incubated in a water bath at 60 °C for 20 min before centrifugation. The supernatant from this procedure was collected to analyze total sugar and proline content.

##### Total Soluble Sugars

The content of soluble sugars was measured using the Antrona method [34]. A 50 μL aliquot of the plant extract was combined with 950 mL of distilled water for this analysis. The tubes were placed in an ice bath to which 2 mL of Antrona was added. After vortexing, the tubes were returned to the ice bath and heated in a water bath for 8 min. Absorbance readings were recorded using a spectrophotometer at a wavelength of 620 nm. The results were reported as milligrams of total soluble sugars (TSSs) per gram of fresh mass.

##### Proline

The quantification of proline was carried out using the method described by Bates et al. (1973) [35]. In this procedure, 1 mL of plant extract, 1 mL of acid ninhydrin, and 1 mL of glacial acetic acid were mixed thoroughly in test tubes. The tubes were then placed in a water bath at 100 °C for 1 h. Afterward, they were cooled in an ice bath, and 2 mL of toluene was added before shaking the tubes. The mixture was then aspirated using a Pasteur pipette, and absorbance was measured with a spectrophotometer at a wavelength of 520 nm. The results were reported as micromoles of proline per gram of fresh mass.

##### Chlorophyll and Carotenoid Content

The chlorophyll and carotenoid content were assessed by weighing 0.2 g of fresh leaf tissue, which was placed in tightly sealed test tubes. To each tube, 10 mL of 80% acetone was added. The tubes were stored in an ultra-freezer for 24 h. Following this incubation, the extracts were transferred to cuvettes for analysis using a spectrophotometer. Absorbance measurements were taken at 645 nm, 652 nm, and 663 nm for chlorophylls [36] and 470 nm for carotenoids [37]. From these readings, the concentrations of chlorophyll a, chlorophyll b, total chlorophyll (a + b), carotenoids, and the ratio of chlorophyll a to b were calculated. The results were reported in milligrams per gram of fresh leaf tissue (mg g^−1^).

#### 2.5.2. Experiment II

In this Experiment, the variables were analyzed similarly to Experiment I. The variables analyzed included germination test, first germination count, average germination speed, germination speed index, root length, shoot length, root dry mass, shoot dry mass, accelerated aging, and electrical conductivity. The methodological procedures followed for these analyses were identical to those described in Experiment I.

### 2.6. Data Analysis

For both Experiment I and Experiment II, the normality of the data was assessed using the Shapiro–Wilk test. In contrast, the Barlett test verified the homogeneity of variance (homoscedasticity) of the residuals. The data from the experiments were subjected to analysis of variance (ANOVA) using the F-test. Experiment I compared the means using the Scott–Knott test at a 5% significance level. For Experiment II, in addition to the Scott–Knott test, Dunnett’s test was employed at a 5% probability level to compare the control treatment with the other treatments. All statistical analyses were performed using the R v.3.2.3. software package [38].

## 3. Results and Discussion

### 3.1. Experiment I

Chemical desiccation can positively or negatively influence cowpea crops, so the answers obtained will depend on the choice of herbicides used, the time of application, and the characteristics inherent to the crop. Significant differences were observed in the germination, first germination count, average germination speed, and germination speed index of cowpea under herbicide application (Table S1). All herbicides applied caused a reduction in the formation of normal seedlings, with the most significant reduction observed for the combination of the herbicides diquat and carfentrazone, which negatively influenced the formation of seedlings, providing 100% of abnormal seedlings (Table 3). Flumioxazin and atrazine reduced normal seedling formation by 57% and 47%, respectively, compared to the control (Table 3). The control treatment provided the highest formation of normal seedlings (Table 3).

The decrease in the formation of normal seedlings demonstrates that the physiological performance of the seeds was reduced [39], which was reflected in the lower germination potential caused by the herbicides flumioxazin, atrazine, and diquat + carfentrazone. The nocturnal application of these treatments may have caused a reduction in the reserves necessary for root and shoot formation, thus causing stress due to the scarcity of photoassimilates for the seed [9]. In contrast, the results obtained by Raisse et al. (2020) [17] in their study of the diurnal application of herbicides in cowpea showed that plants desiccated with carfentrazone, flumioxazin, and diquat produced seeds with a germination percentage above 80%, as did the control without desiccation, which may indicate a lower translocation of these herbicides during the morning. Notably, it is correct to state that the characteristics of physiological quality and mobilized reserves of seeds are expressed in the evaluations made on seedlings [40].

Due to the abnormality in seed germination using diquat + carfentrazone, it was impossible to evaluate the first germination count, average speed, and germination speed index during the experimental period (Table 4). In addition, it was observed that the first germination count (FGC) was negatively influenced when flumioxazin, saflufenacil, and atrazine were used, and the average germination speed (AGS) was reduced when saflufenacil was used (Table 4). All herbicides caused reductions in the germination speed index (GSI), which was lower in plants desiccated with flumioxazin and atrazine, reducing 57% and 47% of the values of this variable compared to the control (Table 4). The highest GSI value obtained was for the control treatment (Table 4).

The first germination count is related to the vigor of the seeds [41]. The reduction in vigor causes problems in the establishment of crops in successive cultivation, as more vigorous seeds find it easier to express their genetic potential [42]. The average germination speed (AGS) reflects the species’ ability to adapt under unfavorable conditions, indicating that seeds with higher AGS are easier to form high-performance seedlings. The results obtained in FGC and AGS demonstrate that herbicides negatively influenced the metabolic activity of seeds, reducing the transport capacity of reserve tissues for cowpea seedling formation [42,43].

The success of the germination parameters (AGS, FGC, and GSI) is entirely related to some essential aspects of the preharvest desiccation of cowpea, such as the time of application and the choice of herbicide [44]. The results obtained demonstrate that the nocturnal application of herbicides reduces the viability of cowpea seeds. This viability is determined by the process of dry mass transfer at maturation, as seeds with greater vigor generally have a greater amount of accumulated reserves and a good energy and nutritional supply to improve physiological quality [45].

Root and shoot length and root and shoot dry mass were influenced by the herbicides applied during the preharvest of cowpea (Table S2). Root length was reduced using all desiccant herbicides, with the highest values observed in the control treatment (Table 5). The length of the shoots (SL) of seedlings was reduced by 35.69% when glufosinate was applied (Table 5). In addition, the combined and isolated application of diquat and flumioxazin reduced up to 21.08% in LS (Table 5). Root dry mass and shoot dry mass were negatively affected using atrazine, decreasing by 40.6% and 43.7%, respectively (Table 5).

Root and shoot length reduction was observed in the desiccation of other crops, such as soybean [46] and wheat [47]. Silva (2020) [48] in his study found that the herbicides that most influenced cowpea seedling root growth were paraquat and saflufenacil. The increase or reduction in root length between treatments demonstrates differences in cowpea seed vigor [49]. In cases of root reduction, there is a more significant number of abnormalities in seedlings [50]. In addition, the oxidative stress provided by herbicides causes reduced cell viability through cell membrane disruption, which may explain the decrease in root growth and the number of abnormal seedlings [51].

The reduction in shoot growth observed may have been due to the mechanism of action of glufosinate ammonium. This compound is an inhibitor of the enzyme glutamine synthetase and promotes, through the depletion of glutamine and glutamate, a dysfunction in the reactions of nitrogen assimilation, which is the main nutrient responsible for vegetative growth [52].

The lower values of RDM and SDM observed in plants desiccated with atrazine report that this herbicide may have reduced the reserves that promote the proper development of cowpea plants [9]. The decrease in seedling dry mass caused by atrazine can be explained by the immediate reduction in the photosynthetic rate caused by this herbicide in the electron chain with the inhibition of photosynthesis, with a decrease in the production of energy and sugars essential for seed formation and consequently in the aerial part of seedlings [53].

The electrical conductivity and accelerated aging test differed significantly with herbicide application at cowpea preharvest (Table S3). The highest electrical conductivity was observed in the seeds submitted to the application of flumioxazin, atrazine, carfentrazone, glufosinate, and diquat, while glyphosate and the diquat + carfentrazone mixture presented lower values compared to the other herbicides (Table 6). Regarding the accelerated aging test (AA), it was observed that diquat and carfentrazone completely affected seed germination and normal seedling formation of cowpea (Table 6). In addition, diquat + flumioxazin, glyphosate, and diquat + carfentrazone caused reductions of 8%, 7%, and 6%, respectively (Table 6).

The electrical conductivity test evaluates the integrity of the cell membrane, which is responsible for the leaching content of sugars and amino acids in the imbibition solution; therefore, the higher the EC and the leachate contents, the greater the disintegration of the membranes and the lower the vigor of the seed [43]. The results demonstrate that the herbicides flumioxazin, atrazine, carfentrazone, and glufosinate destabilized membranes more significantly. These results confirm the statement proposed by Botelho et al. (2016) [54] that herbicides can negatively affect the solute release rate, resulting in uneven seeds with lower GSI.

The AA estimates the storage potential of seeds, which is determined by the field’s maturation [43]. Therefore, cowpea seeds from plants desiccated with diquat and carfentrazone have low storage capacity due to a higher level of deterioration caused by herbicides [55]. Similar results were observed by Raisse et al. (2020) [17], who found in their study on cowpea that the application of diquat for desiccation promotes inferior physiological quality in AA. Therefore, the herbicide chosen for preharvest desiccation can affect the quality of the seeds aiming at a successive crop. According to Pagliarini et al. (2021) [56], chemical desiccation of a metabolically active plant reduces seed quality due to embryonic malformation and reduced vigor, which makes the use of the seed unfeasible. Thus, observing the effects of each herbicide used in desiccation is essential.

**Figure 1:**
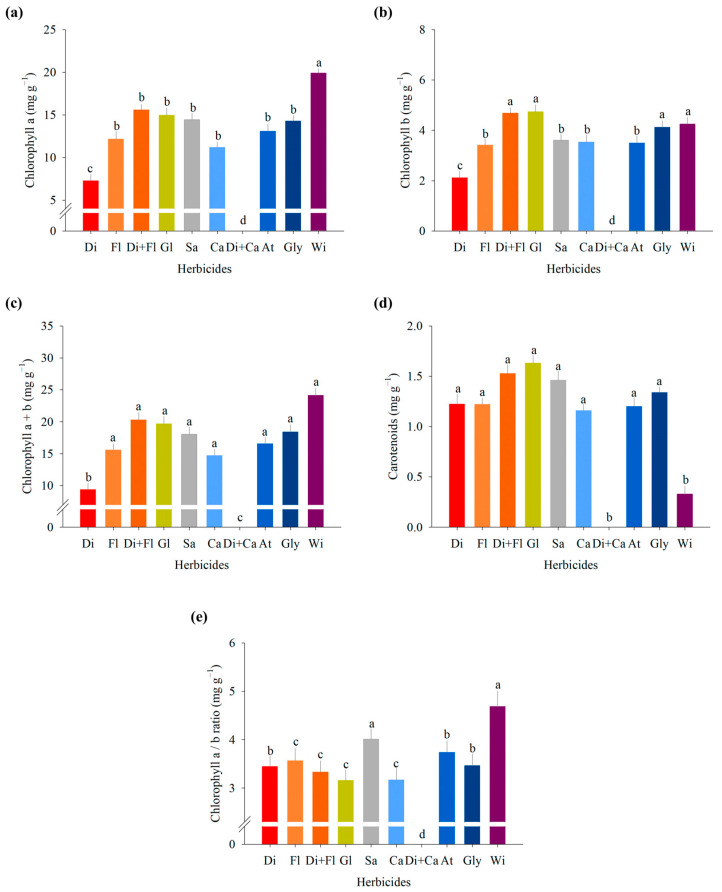
(**a**) Chlorophyll a, (**b**) chlorophyll b, (**c**) chlorophyll a + b, (**d**) carotenoids, and (**e**) ratio between chlorophyll a and chlorophyll b in cowpea seedlings (BRS Tumucumaque) desiccated with herbicides at preharvest. Di: diquat; Fl: flumioxazin; Di + Fl: diquat + flumioxazin; Gl: glufosinate; Sa: saflufenacil; Ca: carfentrazone; Di + Ca: diquat + carfentrazone; At: atrazine; Gly: glyphosate; Wi: witness. Means with the same letter are not significantly different according to the Scott–Knott test at a 5% significance level.

The contents of chlorophyll a, chlorophyll b, and total chlorophyll were negatively influenced by diquat, registering the lowest values (Figure 1a–c). The decrease in chlorophyll levels is an indicator for monitoring seedling development damage [57]. The results obtained for chlorophyll a and b contents confirm the interference of diquat in vital plant processes, which, through the reduction in chlorophyll content, provided a decrease in the levels of sugars essential for the development of seedlings, since a decrease in photosynthetic pigments is directly related to a reduction in photosynthesis [58].

For carotenoids, increases were observed with herbicides, while the control showed the lowest value (Figure 1d). The increase in carotenoids with the application of herbicides reflects its function as an accessory pigment for light capture and as an essential agent for protection against photo-oxidation caused by chlorophyll in its triplet state [59,60], which is characterized as a non-enzymatic antioxidant compound [61]. According to Salem and Sobki (2021) [62], herbicides can positively or negatively affect the formation of chloroplasts, thus causing an increase or decrease in photosynthetic pigments, where the plant’s susceptibility is influenced by the mode of action of each herbicide, as well as by the carotenoid values found.

The chlorophyll a and b ratio showed the lowest values using glufosinate, carfentrazone, diquat + flumioxazin, diquat, and glyphosate (Figure 1e). The decrease in chlorophyll observed in studies with herbicides in cowpea can be explained by the increase in the expression of the CHLASE gene, which encodes the enzyme chlorophyllase and promotes increased chlorophyll degradation through the activity of this enzyme [63]. The results obtained for chlorophyll a and b and for the ratio between chlorophyll a and b further elucidate the function of carotenoids as antioxidants under conditions of stress caused by herbicides, with the increase in their biosynthesis [64,65]. In the present study, carotenoids increased due to a reduction in chlorophyll a and b contents.

**Figure 2:**
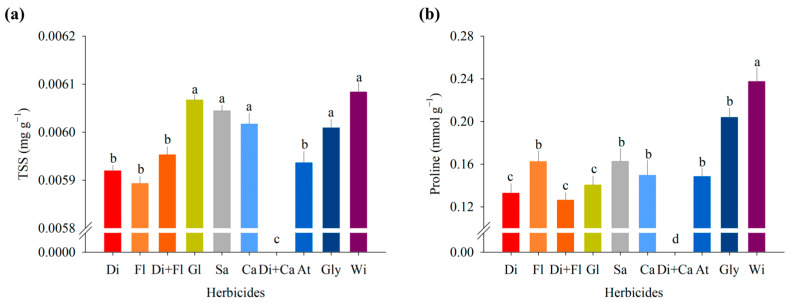
(**a**) Total soluble sugars (TSSs) and (**b**) proline in cowpea seedlings (BRS Tumucumaque) desiccated with herbicides at preharvest. Di: diquat; Fl: flumioxazin; Di + Fl: diquat + flumioxazin; Gl: glufosinate; Sa: saflufenacil; Ca: carfentrazone; Di + Ca: diquat + carfentrazone; At: atrazine; Gly: glyphosate; Wi: witness. Means with the same letter are not significantly different according to the Scott–Knott test at a 5% significance level.

The contents of total soluble sugars (TSSs) and proline were influenced by herbicides applied during the preharvest of cowpea (Figure 2a,b). Total soluble sugars decreased when flumioxazin and diquat were applied, with 0.00589 and 0.00591 mg g^−1^, respectively, while in control, these values were 0.00608 (Figure 2a). In addition, it was also possible to observe a decrease in the values of sugars when the mixture of diquat + flumioxazin was applied (Figure 2a). Flumioxazin works by inhibiting the enzyme protoporphyrinogen oxidase (PROTOX), which is responsible for catalyzing the conversion of protoporphyrinogen IX to protoporphyrin IX in chlorophyll biosynthesis [66]. The decrease in sugars caused by desiccation with diquat can be explained by the reduction in chlorophyll a and b caused by this herbicide in the present study, thus reflecting a dysfunctionality in the photosynthetic process since this herbicide is responsible for the inhibition of PSI, functioning as an electron acceptor in the photochemical stage of photosynthesis.

### 3.2. Experiment II

The interaction between application times and herbicides was significant for the first germination count (FGC) and germination speed index (GSI) (Table S4). Regarding germination, the factors (schedules and herbicides) were significant in isolation, while the average germination speed differed significantly between the application times. The control treatment differed significantly from the other treatments for germination, first germination count, and germination speed index.

The application of diquat, diquat + carfentrazone, and diquat + flumioxazin at all times caused a reduction in cowpea seed germination compared to the control without herbicide (Table 7). The application of herbicides at 12 p.m. provided the lowest germination values. At this time, the herbicides diquat + carfentrazone and diquat + flumioxazin caused the highest formation of abnormal seedlings (Table 7). A higher incidence of dead seeds was observed with the application of diquat at 12 p.m. (Table 7). The condition that promoted higher germination values and, consequently, lower formation of abnormal seedlings occurred at 6 a.m. (Table 7).

The decrease in germination reflects the reduction in germination potential caused by applying herbicides in preharvest desiccation, demonstrating that using these herbicides for cowpea seed production compromises physiological quality [54]. The effects of herbicide application on desiccated crops may be associated with changes in seed germination metabolism through the reduction in essential reserves for the formation of normal seedlings [9]. The results observed at the time of application show that the possible heat stress provided to the plants in the application condition at 12 p.m. increased the damage caused by the herbicides [67]. According to Barrozo et al. (2020) [68], high temperatures at hotter times of the day can cause irreversible damage to plants, interfering with the physiological quality of seeds. In addition, the variation of environmental factors such as relative humidity, hours of exposure to light, and temperature can directly influence the effectiveness and effects of the herbicides applied [69].

The application of herbicides at all times provided a decrease in the values of the first germination count (FGC), average germination speed (AGS), and germination speed index (GSI) (Table 8). The first germination count (FGC) was lower with the application of diquat and diquat + flumioxazin at 12 p.m., with a reduction of 70% (Table 8). The application of diquat + carfentrazone caused a similar reduction between 12 p.m. and 6 p.m. for the first germination count (Table 8). Applying diquat + flumioxazin at 12 p.m. resulted in the lowest value for the average germination speed (AGS), which was reduced by 22.5% (Table 8). The results obtained for the average germination speed also demonstrate the reduction in seed vigor with the application of diquat + flumioxazin at 12 p.m. (Table 8). The germination speed index (GSI) was reduced by applying diquat + carfentrazone and diquat + flumioxazin at 12 p.m. (Table 8). In addition, diquat + flumioazin applied at 6 p.m. reduced GSI by 44.5% (Table 8).

The reduction observed in the first germination count demonstrates the decrease in vigor caused by applying diquat and diquat + flumioxazin at 12 p.m. [70]. Seeds with low vigor result in a more excellent formation of abnormal seedlings and compromise the establishment of the stand in cases of use of this seed for successive cultivation [42,71]. Therefore, the results show that applying diquat and diquat + flumioxazin reduces the vigor of cowpea seeds. Thus, in addition to factors such as health, longevity, environmental conditions, and genetic characteristics, it should be considered that the application of herbicides associated with the choice of application time influences the ability of seeds to originate normal seedlings.

A low average germination speed is an indication of abnormality in seedlings and may be a determining factor for crop establishment in successive cultivation situations [72]. The average germination speed is related to the time scale of water absorption during germination, which is determined by the genotype used in the crop, the germination conditions, and the reserves accumulated during grain filling [72]. The results obtained for AGS and GSI may indicate that the environmental conditions at the application times (12 p.m. and 6 p.m.) negatively influenced germination potential expression through herbicides’ more evident action [73,74]. Notably, the different results for the germination parameters show an effect intensity due to the different application times.

The interaction between application times and herbicides was significant for root length (RL) and shoot dry mass (SDM) (Table S5). Regarding root length (RL), there was significance for the application of herbicides in isolation, while shoot length was significant for both factors (application times and herbicides) (Table S5). The dry mass of the aerial part was also significant in isolation for the herbicides and time of application, as well as for the interaction between them (Table S5). The control treatment differed significantly from the other treatments in terms of root length, shoot length, and root dry mass (Table S5).

The lowest root length value was found with the nocturnal application of diquat + flumioxazin, with a reduction of 48.19% (Table 9). In addition, diquat + sulfentrazone at 12 p.m. provided a reduction in root length of 38.64%. The application of diquat at the three application times (6 a.m., 12 p.m., and 6 p.m.) did not differ from the control treatment in terms of root length.

The lowest value found for root length was with the nocturnal application of diquat + flumioxazin, with a reduction of 48.19% (Table 10). In addition, diquat + carfentrazone at 12 p.m. reduced root length by 38.64% (Table 10). The application of diquat at the three application times (6 a.m., 12 p.m., and 6 p.m.) did not differ from the control treatment in terms of root length (Table 10). Shoot length was reduced with the application of herbicides at all application times, with a decrease of up to 30.18% (Table 10). However, the application of diquat + carfentrazone at 6 a.m. did not differ from the control treatment in terms of shoot length (Table 10). Decreases in root dry mass (RDM) and shoot mass (SDM) were observed with the nocturnal application of diquat + flumioxazin (Table 10).

The results obtained for root length demonstrate that applications at 12 p.m. and 6 p.m. caused more damage to seedling root development. However, other authors confirm that the application of herbicides during preharvest causes a reduction in seedling development, regardless of the time of application [8,75]. Decreases in root and shoot length are directly related to the formation of abnormal seedlings [50]. Therefore, a reduction in seedling growth is a result of the effects caused by herbicides, since the application of desiccants caused a more excellent formation of abnormal seedlings. According to Mahapatra et al. (2019) [51], applying herbicides can simulate a stress condition for plants, which reduces their cell viability by disrupting membranes and interfering with the development of seeds and seedlings. The observed differences in the effects caused by herbicides due to application schedules may indicate that the same herbicide may have a variable efficiency depending on environmental factors at the time of application [76]. The decrease in dry mass may be associated with a reduction in essential sugars and reserves required to form normal seedlings [53].

The interaction between application times and herbicides was significant for accelerated aging and electrical conductivity (Table S6). For electrical conductivity (EC), significance was observed in isolation for the application of desiccants and the application times, while accelerated aging was significant for the application times (Table S6). The control differed significantly from the other treatments in terms of accelerated aging and electrical conductivity (Table S6).

The application of diquat and diquat + flumioxazin at 12 p.m. provided a reduction in the values of the accelerated aging test by 35.52% and 39.46%, respectively (Table 11). The highest values of electrical conductivity (EC) were obtained with the application of diquat and diquat + carfentrazone at 12 p.m., which caused an increase of 20.28% and 28.3%, respectively (Table 11). The other treatments were similar to the control (Table 11).

Accelerated aging simulates adverse conditions to estimate the storage potential of seeds [43]. Therefore, the results obtained for AA indicate that cowpea seeds from plants desiccated with diquat and diquat + flumioxazin may have low storage potential, which compromises the use of this seed for successive planting. The higher values found for electrical conductivity indicate that plants subjected to desiccation with diquat and diquat + carfentrazone, presented seeds with greater intensity of membrane damage. Thus, the rate of solute release directly influences the quality of the seeds, as the leachate output results in a loss of vigor [77].

Overall, this study’s results demonstrate that the timing of herbicide application significantly influences cowpea seed quality, physiological responses, and biochemical composition. The reductions in chlorophyll levels, increase in carotenoid content, and impacts on soluble sugars and proline highlight the physiological stress induced by herbicide treatments. Notably, nocturnal desiccation led to the most severe alterations in seedling physiology, suggesting that herbicide translocation and its effects vary according to application time.

## 4. Conclusions

The results show the importance of studies that address the impacts caused by herbicides on the physiological quality of seeds. Although the results may indicate a distinct translocation between application times, research that can confirm the translocation of herbicides to the seed is necessary. The results obtained are of great importance for the cultivation of cowpea and can be used as a scientific basis to increase the effectiveness of herbicide use.

The different application times affect the physiological quality of cowpea seeds differently. The herbicides at all application times reduced seed germination and vigor, as well as initial seedling growth. The use of the associations between diquat + carfentrazone and diquat + flumioxazin causes more severe effects, and the application at night and 12 p.m. intensifies the damage caused by these desiccants.

## Figures and Tables

**Table 1:** Chemical properties of the soil at depths of 20 cm and 40 cm in the experimental site.

	pH in H_2_O	EC	P	K^+^	Na^+^	Ca^2+^	Mg^2+^
		dS m^−1^	mg dm^−3^			cmol_c_ dm^−3^	
20 cm	7.56	0.08	156.77	156.00	15.20	3.50	0.86
40 cm	7.45	0.05	106.23	145.87	15.20	3.00	0.43

EC: electrical conductivity.

**Table 2:** Characterization and concentrations of herbicides.

Active Ingredient	Commercial Product	Commercial Product Dose	Applied Dose of Active Ingredient (a.i.)/Acid Equivalent (a.e.)
Diquat	Reglone	2.0 L ha^−1^	400.00 g a.i. ha^−1^
Flumioxazin	Sumyzin	50 mL ha^−1^	25.00 g a.i. ha^−1^ *
Diquat + flumioxazin	Reglone + Sumyzin500	2.0 L ha^−1^ + 50 mL ha^−1^	400.00 g a.i. ha^−1^ + 25.00 g a.i. ha^−1^ *
Glufosinate	Fascinate BR	2.0 L ha^−1^	400.00 g a.i. ha^−1^
Saflufenacil	Heat	140 g ha^−1^	98.00 g a.i. ha^−1^
Carfentrazone-ethyl	Aurora	125 mL ha^−1^	50.00 g a.i. ha^−1^ *
Diquat + carfentrazone	Reglone + Aurora	2.0 L ha^−1^ + 125 mL ha^−1^	400.00 g a.i. ha^−1^ + 50.00 g a.i. ha^−1^ *
Atrazine	Herbitrin	5.0 L ha^−1^	2.50 g a.i. ha^−1^ *
Glyphosate	Roundup original DI	4.0 L ha^−1^	1.480 g a.e. ha^−1^

* Addition of 0.5% *v*/*v* of mineral oil.

**Table 3:** Normal seedlings, abnormal seedlings, and dead seeds of cowpea (BRS Tumucumaque) subjected to preharvest herbicide application.

Herbicides	Normal Seedlings (%)	Abnormal Seedlings (%)	Dead Seeds (%)
Diquat	49 b	49 c	2 a
Flumioxazin	35 c	64 b	0 a
Diquat + flumioxazin	55 b	44 c	1 a
Glufosinate	59 b	40 c	1 a
Saflufenacil	51 b	48 c	0 a
Carfentrazone	49 b	48 c	2 a
Diquat + carfentrazone	0 d	100 a	0 a
Atrazine	43 c	55 b	1 a
Glyphosate	56 b	43 c	0 a
Witness (control)	82 a	17 d	0 a
CV (%)	7.80	7.91	6.50

Means with the same letter in the column are not significantly different according to the Scott–Knott test at a 5% significance level. CV: coefficient of variation.

**Table 4:** First germination count (FGC), average germination speed (AGS), and germination speed index (GSI) of cowpea plant seeds (BRS Tumucumaque) subjected to preharvest herbicide application.

Herbicides	FGC	AGS	GSI
Diquat	45 b ± 4.96	0.3211 a ± 0.028	7.962 b ± 1.23
Flumioxazin	33 c ± 5.78	0.3218 a ± 0.035	5.787 c ± 0.87
Diquat + flumioxazin	52 b ± 4.38	0.3288 a ± 0.026	8.383 b ± 1.04
Glufosinate	55 b ± 4.66	0.3259 a ± 0.028	9.662 b ± 1.28
Saflufenacil	36 c ± 5.71	0.2876 b ± 0.023	7.712 b ± 1.23
Carfentrazone	47 b ± 5.12	0.3258 a ± 0.023	8.120 b ± 1.21
Diquat + carfentrazone	0 d ± 0.00	0.0000 c ± 0.000	0.000 d ± 0.00
Atrazine	40 c ± 5.05	0.3206 a ± 0.027	7.066 c ± 1.14
Glyphosate	52 b ± 5.12	0.3231 a ± 0.025	9.129 b ± 1.21
Witness (control)	76 a ± 6.15	0.3236 a ± 0.030	13.466 a ± 1.81

Means with the same letter in the column are not significantly different according to the Scott–Knott test at a 5% significance level. Number after the mean represents the standard error (*n* = 4 replications).

**Table 5:** Root length (RL), shoot length (SL), root dry mass (RDM), and shoot dry mass (SDM) of cowpea seedlings (BRS Tumucumaque) desiccated with herbicides at preharvest.

Herbicides	RL (cm Seedling^−1^)	SL (cm Seedling^−1^)	RDM (g Seedling^−1^)	SDM (g Seedling^−1^)
Diquat	8.81 b ± 1.12	6.65 c ± 0.85	0.30 a ± 0.04	2.53 a ± 0.25
Flumioxazin	9.32 b ± 1.08	6.48 c ± 0.78	0.20 b ± 0.06	1.90 b ± 0.27
Diquat + flumioxazin	9.22 b ± 1.15	6.65 c ± 0.82	0.22 b ± 0.05	2.28 a ± 0.27
Glufosinate	9.07 b ± 1.10	5.28 c ± 0.90	0.24 b ± 0.05	2.47 a ± 0.26
Saflufenacil	8.73 b ± 1.05	7.38 b ± 0.80	0.23 b ± 0.05	1.98 b ± 0.28
Carfentrazone	9.72 b ± 1.20	7.36 b ± 0.75	0.24 b ± 0.06	1.91 b ± 0.29
Diquat + carfentrazone	0.00 c ± 0.00	0.00 d ± 0.00	0.00 d ± 0.00	0.00 d ± 0.00
Atrazine	9.89 b ± 1.18	7.31 b ± 0.85	0.19 c ± 0.05	1.59 c ± 0.27
Glyphosate	10.20 b ± 1.22	7.21 b ± 0.80	0.29 a ± 0.06	2.33 a ± 0.28
Witness	11.31 a ± 1.25	8.21 a ± 0.90	0.32 a ± 0.05	2.82 a ± 0.30

Means with the same letter in the column are not significantly different according to the Scott–Knott test at a 5% significance level. Number after the mean represents the standard error (*n* = 4 replications).

**Table 6:** Electrical conductivity (EC) and accelerated aging test (AA) of cowpea plant seeds (BRS Tumucumaque) subjected to preharvest herbicide application.

Herbicides	EC (μS cm^−1^g^−1^)	AA
Diquat	22.61 a ± 1.50	0 c ± 0.00
Flumioxazin	24.43 a ± 1.75	98 a ± 2.50
Diquat + flumioxazin	22.85 a ± 1.60	92 b ± 3.00
Glufosinate	23.51 a ± 1.80	99 a ± 2.00
Saflufenacil	21.91 a ± 1.55	96 a ± 2.75
Carfentrazone	23.81 a ± 1.70	0 c ± 0.00
Diquat + carfentrazone	19.71 b ± 1.45	94 b ± 3.25
Atrazine	24.35 a ± 1.65	96 a ± 2.50
Glyphosate	19.96 b ± 1.50	93 b ± 3.00
Witness	16.89 c ± 1.85	100 a ± 1.75

Means with the same letter in the column are not significantly different according to the Scott–Knott test at a 5% significance level. Number after the mean represents the standard error (*n* = 4 replications).

**Table 7:** Normal seedlings, abnormal seedlings, and dead seeds of cowpea plants (BRS Tumucumaque) were subjected to preharvest herbicide application at different times.

Variables	Herbicides	Times (h)		
		6 a.m.	12 p.m.	6 p.m.
Normal seedlings (%)	Diquat	47.7 bA	38.8 aB	46.0 aA
	Diquat + carfentrazone	56.5 aA	39.0 aC	46.5 aB
	Diquat + flumioxazin	47.0 bA	35.5 aB	44.5 aA
	Witness	67.0 α		
	CV (%)	7.95		
Abnormal seedlings (%)	Diquat	45.2 bA	47.5 bA	50.2 aA
	Diquat + carfentrazone	41.0 bC	57.0 aA	50.5 aB
	Diquat + flumioxazin	51.0 aA	55.7 aA	52.5 aA
	Witness	31.5 α		
	CV (%)	7.93		
Dead seeds (%)	Diquat	7.0 aB	14.0 aA	3.7 aB α
	Diquat + carfentrazone	2.5 bA α	4.0 cA α	3.0 aA α
	Diquat + flumioxazin	2.0 bB α	8.7 bA	3.0 aB α
	Witness	1.5 α		
	CV (%)	9.01		

CV: coefficient of variation; means followed by the same lowercase letters in the columns and means followed by the same uppercase letters in the rows do not differ from each other by the Scott–Knott test at 5% probability; means followed by “α” do not differ from the control by the Dunnett test at a 5% probability.

**Table 8:** First germination count (FGC), average germination speed (AGS), and germination speed index (GSI) of seeds from cowpea plants (BRS Tumucumaque) subjected to preharvest herbicide application at different times.

Variables	Herbicides	Times (h)		
		6 a.m.	12 p.m.	6 p.m.
FGC (%)	Diquat	14.5 aA	7.5 bC	11.5 bB
	Diquat + carfentrazone	14.5 aA	10.5 aB	10.5 bB
	Diquat + flumioxazin	14.5 aA	7.5 bB	13.0 aA
	Witness	25.0 α		
AGS (days)	Diquat	0.5356 aA α	0.4703 aB α	0.4484 aB α
	Diquat + carfentrazone	0.5721 aA α	0.4666 aB α	0.4934 aB α
	Diquat + flumioxazin	0.5089 aA α	0.4057 aB	0.4945 aA α
	Witness	0.5234 α		
GSI	Diquat	12.46 aA	13.37 aA	13.62 aA
	Diquat + carfentrazone	14.35 aA	10.91 aB	13.45 aA
	Diquat + flumioxazin	14.24 aA	10.83 aB	11.87 aA
	Witness	21.37 α		

Means followed by the same lowercase letters in the columns and means followed by the same uppercase letters in the rows do not differ from each other by the Scott–Knott test at 5% probability; means followed by “α” do not differ from the control by the Dunnett test at a 5% probability.

**Table 9:** Root length (RL), shoot length (SL), root dry mass (RDM), and shoot dry mass (SDM) of cowpea seedlings (BRS Tumucumaque) desiccated with herbicides at preharvest at different times.

Variables	Herbicides	Times (h)		
		6 a.m.	12 p.m.	6 p.m.
RL (cm seedling^−1^)	Diquat	9.345 aA α	9.624 aA α	10.606 aA α
	Diquat + carfentrazone	10.853 aA α	7.229 aB	8.530 bA
	Diquat + flumioxazin	8.212 bA	8.762 aA	6.103 bB
	Witness	11.781 α		
SL (cm seedling^−1^)	Diquat	7.142 aA	6.325 aA	6.365 aA
	Diquat + carfentrazone	7.415 aA α	6.361 aB	6.105 aB
	Diquat + flumioxazin	6.197 bA	5.661 aA	6.153 aA
	Witness	8.108 α		
RDM (g seedling^−1^)	Diquat	0.0132 aA	0.0121 aA	0.0138 aA
	Diquat + carfentrazone	0.0127 aA	0.0125 aA	0.0144 aA α
	Diquat + flumioxazin	0.0129 aA	0.0114 aA	0.0105 bB
	Witness	0.0175 α		
SDM (g seedling^−1^)	Diquat	0.1460 aA α	0.1468 aA α	0.1406 aA α
	Diquat + carfentrazone	0.1496 aA α	0.1249 bB	0.1371 bA α
	Diquat + flumioxazin	0.1438 aA α	0.1418 aA α	0.1254 bB
	Witness	0.1459 α		

Means followed by the same lowercase letters in the columns and means followed by the same uppercase letters in the rows do not differ from each other by the Scott–Knott test at 5% probability; means followed by “α” do not differ from the control by the Dunnett test at a 5% probability.

**Table 10:** Root length (RL), shoot length (SL), root dry mass (RDM), and shoot dry mass (SDM) of cowpea seedlings (BRS Tumucumaque) desiccated with herbicides at preharvest at different times.

Variables	Herbicides	Times (h)		
		6 a.m.	12 p.m.	6 p.m.
RL (cm seedling^−1^)	Diquat	9.345 aA α	9.624 aA α	10.606 aA α
	Diquat + carfentrazone	10.853 aA α	7.229 aB	8.530 aB
	Diquat + flumioxazin	8.212 aA	8.762 aA	6.103 bB
	Witness	11.781 α		
SL (cm seedling^−1^)	Diquat	7.142 aA	6.325 aA	6.365 aA
	Diquat + carfentrazone	7.415 aA α	6.361 aB	6.105 aB
	Diquat + flumioxazin	6.197 bA	5.661 aA	6.153 aA
	Witness	8.108 α		
RDM (g seedling^−1^)	Diquat	0.0132 aA	0.0121 aA	0.0138 aA
	Diquat + carfentrazone	0.0127 aA	0.0125 aA	0.0144 aA α
	Diquat + flumioxazin	0.0129 aA	0.0114 aB	0.0105 bB
	Witness	0.0175 α		
SDM (g seedling^−1^)	Diquat	0.1460 aA α	0.1468 aA α	0.1406 aA α
	Diquat + carfentrazone	0.1496 aA α	0.1249 bB	0.1371 bA α
	Diquat + flumioxazin	0.1438 aA α	0.1418 aA α	0.1254 bB
	Witness	0.1459 α		

Means followed by the same lowercase letters in the columns and means followed by the same uppercase letters in the rows do not differ from each other by the Scott–Knott test at 5% probability; means followed by “α” do not differ from the control by the Dunnett test at a 5% probability.

**Table 11:** Accelerated aging (AA) and electrical conductivity (EC) tests of cowpea plant seeds (BRS Tumucumaque) subjected to preharvest herbicide application at different times.

Variables	Herbicides	Times (h)		
		6 a.m.	12 p.m.	6 p.m.
AA (%)	Diquat	40.5 aA	29.5 bB	38.7 aA
	Diquat + carfentrazone	39.5 aA	33.7 aB	33.7 bB
	Diquat + flumioxazin	40.0 aA	27.7 bC	35.2 bB
	Witness	45.75 α		
EC (μS cm^−1^g^−1^)	Diquat	72.46 aB	83.61 bA	75.18 aB
	Diquat + carfentrazone	72.56 aB	92.97 bA	78.90 aB
	Diquat + flumioxazin	75.46 aA	76.90 aA	78.58 aA
	Witness	66.66 α		

Means followed by the same lowercase letters in the columns and means followed by the same uppercase letters in the rows do not differ from each other by the Scott–Knott test at 5% probability; means followed by “α” do not differ from the control by the Dunnett test at a 5% probability.

## Data Availability

Data are contained within the article and Supplementary Materials.

## Supplementary Materials

The following supporting information can be downloaded at https://www.mdpi.com/article/10.3390/jox14030074/s1, Table S1: Analysis of variance of germination (G), first germination count (FGC), average germination speed (AGS), and germination speed index (GSI) of cowpea plant seeds (BRS Tumucumaque) subjected to preharvest herbicide application; Table S2: Analysis of variance of root length (RL), shoot length (SL), root dry mass (RDM), and shoot dry mass (SDM) of cowpea seedlings (BRS Tumucumaque) desiccated with herbicides at preharvest; Table S3: Analysis of variance of electrical conductivity (EC) and accelerated aging test (AA) of cowpea plant seeds (BRS Tumucumaque) subjected to preharvest herbicide application; Table S4: Analysis of variance of germination (G), first germination count (FGC), average germination speed (AGS), and germination speed index (GSI) of seeds from cowpea plants (BRS Tumucumaque) subjected to herbicide application at preharvest at different times; Table S5: Analysis of variance of root length (RL), shoot length (SL), root dry mass (RDM), and shoot dry mass (SDM) of cowpea seedlings (BRS Tumucumaque) desiccated with herbicides at preharvest at different times; Table S6: Analysis of variance of the accelerated aging (AA) test and electrical conductivity (EC) in cowpea plant seeds (BRS Tumucumaque) subjected to preharvest herbicide application at different times.
