# Supplementary material for: Time of Application of Desiccant Herbicides Affects Photosynthetic Pigments, Physiological Indicators, and the Quality of Cowpea Seeds
Source: J Xenobiot. 2024 Sep 19;14(3):1312–31. doi: 10.3390/jox14030074 (PMC11417823; doi:10.3390/jox14030074)
Supplement: Supplementary file 1 [file jox-14-00074-s001.zip › Table S1.pdf]

**Table S1.** Analysis of variance of germination (G), first germination count (FGC), average germination speed (AGS), and germination speed index (GSI) of cowpea plant seeds (BRS Tumucumaque) subjected to preharvest herbicide application.

| Sources of variation | F test | | | |
|---|---|---|---|---|
| | G | FGC | AGS | GSI |
| Herbicides | 52.53** | 43.73** | 330.39** | 49.07** |
| Mean | 48.15 | 43.80 | 0.29 | 7.73 |
| CV (%) | 7.80 | 8.97 | 3.89 | 7.34 |

**: significant at 1% probability by F test;
CV: coefficient of variation.
