# Supplementary material for: Time of Application of Desiccant Herbicides Affects Photosynthetic Pigments, Physiological Indicators, and the Quality of Cowpea Seeds
Source: J Xenobiot. 2024 Sep 19;14(3):1312–31. doi: 10.3390/jox14030074 (PMC11417823; doi:10.3390/jox14030074)
Supplement: Supplementary file 1 [file jox-14-00074-s001.zip › Table S2.pdf]

Table S2. Analysis of variance of root length (RL), shoot length (SL), root dry mass (RDM), and shoot dry mass (SDM) of cowpea seedlings (BRS Tumucumaque) desiccated with herbicides in preharvest.

Sources of variation	F test			
	RL	SL	RDM	SDM
Herbicides	48.18**	138.11**	12.40**	11.15**
Mean	8.63	6.00	0.22	1.98
CV (%)	7.07	6.39	5.23	8.61

**: significant at 1% probability by F test;
CV: coefficient of variation.
