# Supplementary material for: Time of Application of Desiccant Herbicides Affects Photosynthetic Pigments, Physiological Indicators, and the Quality of Cowpea Seeds
Source: J Xenobiot. 2024 Sep 19;14(3):1312–31. doi: 10.3390/jox14030074 (PMC11417823; doi:10.3390/jox14030074)
Supplement: Supplementary file 1 [file jox-14-00074-s001.zip › Table S3.pdf]

Table S3. Analysis of variance of electrical conductivity (EC) and accelerated aging test (AA) of cowpea plant seeds (BRS Tumucumaque) subjected to preharvest herbicide application.

Sources of variation	F test	
	EC	AA
Herbicides	12.20**	787.25**
Mean	22.00	77.00
CV (%)	6.35	3.76

**: significant at 1% probability by F test;
CV: coefficient of variation.
