# Supplementary material for: Time of Application of Desiccant Herbicides Affects Photosynthetic Pigments, Physiological Indicators, and the Quality of Cowpea Seeds
Source: J Xenobiot. 2024 Sep 19;14(3):1312–31. doi: 10.3390/jox14030074 (PMC11417823; doi:10.3390/jox14030074)
Supplement: Supplementary file 1 [file jox-14-00074-s001.zip › Table S4.pdf]

Table S4. Analysis of variance of germination (G), first germination count (FGC), average germination speed (AGS), and germination speed index (GSI) of seeds from cowpea plants (BRS Tumucumaque) subjected to herbicide application in preharvest at different times.

Sources of variation	F test			
	G	FGC	AGS	GSI
Times (T)	35.95 **	67.56 **	11.67 **	5.50 **
Herbicides (H)	5.57 **	0.90 ns	2.32 ns	1.00 ns
T x H	1.91ns	5.27 **	1.55 ns	3.28 *
Witness x Factorial	130.53 **	406.69 **	1.99 ns	120.83 **
CV (%)	7.95	8.80	9.56	9.84

CV: coefficient of variation;
**: significant at 1% probability according to the F test;
*: significant at 5% probability according to the F test;
ns: not significant.
