# Supplementary material for: Time of Application of Desiccant Herbicides Affects Photosynthetic Pigments, Physiological Indicators, and the Quality of Cowpea Seeds
Source: J Xenobiot. 2024 Sep 19;14(3):1312–31. doi: 10.3390/jox14030074 (PMC11417823; doi:10.3390/jox14030074)
Supplement: Supplementary file 1 [file jox-14-00074-s001.zip › Table S6.pdf]

Table S6. Analysis of variance of the accelerated aging (AA) test and electrical conductivity (EC) in cowpea plant seeds (BRS Tumucumaque) subjected to preharvest herbicide application at different times.

Sources of variation	F test	
	AA	EC
Times (T)	60.77 **	11.24 **
Herbicides (H)	2.49 ^{ns}	5.11 *
T x H	5.78 **	4.62 **
Witness x Factorial	82.66 **	4.63 *
CV (%)	5.91	8.43

CV: coefficient of variation;
**: significant at 1% probability by F test;
*: significant at 5% probability by F test;
^{ns}: not significant.
